# Discharge Navigator: Implementation and Cross-Sectional Evaluation of a Digital Decision Tool for Social Resources upon Emergency Department Discharge

**DOI:** 10.5811/westjem.2022.5.55015

**Published:** 2022-08-11

**Authors:** Madeline Grade, Nicholas Stark, David Emanuels, Alice Lu, Jay Doshi, Sherman Leung, Christopher Peabody

**Affiliations:** * University of California San Francisco, Department of Emergency Medicine, San Francisco, California; † University of California San Francisco School of Medicine, San Francisco, California; ‡ Cornell University, Ithaca, New York; § Icahn School of Medicine at Mount Sinai, New York City, New York; ¶ University of California San Francisco, Acute Care Innovation Center, San Francisco, California

## Abstract

**Introduction:**

Many patients have unaddressed social needs that significantly impact their health, yet navigating the landscape of available resources and eligibility requirements is complex for both patients and clinicians.

**Methods:**

Using an iterative design-thinking approach, our multidisciplinary team built, tested, and deployed a digital decision tool called “Discharge Navigator” (edrive.ucsf.edu/dcnav) that helps emergency clinicians identify targeted social resources for patients upon discharge from the acute care setting. The tool uses each patient’s clinical and demographic information to tailor recommended community resources, providing the clinician with action items, pandemic restrictions, and patient handouts for relevant resources in five languages. We implemented two modules at our urban, academic, Level I trauma center.

**Results:**

Over the 10-week period following product launch, between 4–81 on-shift emergency clinicians used our tool each week. Anonymously surveyed clinicians (n = 53) reported a significant increase in awareness of homelessness resources (33% pre to 70% post, P<0.0001) and substance use resources (17% to 65%, P<0.0001); confidence in accessing resources (22% to 74%, P<0.0001); knowledge of eligibility criteria (13% to 75%, P<0.0001); and ability to refer patients always or most of the time (11% to 43%, P<0.0001). The average likelihood to recommend the tool was 7.8 of 10.

**Conclusion:**

Our design process and low-cost tool may be replicated at other institutions to improve knowledge and referrals to local community resources.

## BACKGROUND

The field of emergency medicine (EM) recognizes that emergency care extends beyond meeting patients’ acute medical needs; addressing patients’ underlying psychosocial needs is a key tenet of social EM.^1^–^3^ Considering the complex medical, behavioral, and social needs of individual patients is vital to provide well-rounded care that addresses structural determinants of health such as racism and poverty.^4^–^6^ Such an approach necessitates both attentive care within the emergency department (ED) and connecting patients with community resources upon discharge. However, the complexity of navigating available resources is a barrier that may leave social needs unaddressed.

Several companies have attempted to tackle this challenge by developing electronic databases, search tools, and community referral platforms with the goal of connecting patients to social resources. Widely used platforms include 1Degree (San Francisco CA), Unite Us (New York, NY), and Aunt Bertha (now findhelp.org) (Austin, TX).^7^ Most of these tools integrate a resource directory with a referral tracking component and offer some degree of filtering by category of patient need. However, these platforms are often patient-facing and tend to present patients an overwhelming number of potential resources, which can be time-consuming and painstaking for patients and clinicians to sift through without aid from a social work team. Moreover, only a few provide patients with translated materials in Spanish and even fewer offer any other languages, which is an important gap given our diverse patient population. The existing tools did not meet our need for a targeted list of local resources tailored to specific patient needs. We were also looking for the flexibility to customize listings and prioritize institution-specific resource recommendations, as well as embed clinician action items per resource to facilitate the referral process.

## OBJECTIVES

Using an iterative design-thinking approach, our team aimed to create a digital decision tool to help clinicians identify and link patients to social resources upon discharge. We sought to make this tool 1) customizable, using each patient’s clinical and demographic information to tailor recommended local resources, and 2) actionable, providing the clinician with clear next steps, patient handouts in multiple languages, and updated pandemic restrictions. We also aimed to evaluate the impact of this tool on clinicians’ knowledge and confidence in caring for patients with discharge needs in domains such as housing and substance use. Ultimately, we intended to augment the existing institutional processes for patient referrals (social work, social medicine team). Through this intervention, we hoped to fortify an institutional culture of addressing social needs at multiple levels of clinical care.

## DESIGN

### Setting the Stage for Innovation

Our institution, San Francisco General Hospital, is a Level I trauma center with academic affiliations with the University of California San Francisco (UCSF). Prior to building our tool, we determined key stakeholders among patients, hospital and department leadership, and community partners. We also explored available funding and logistical resources to ensure sustainability. We housed this project within the UCSF Department of Emergency Medicine’s Acute Care Innovation Center (acutecare.ucsf.edu) and obtained departmental support for implementing a new tool in our clinical workflow.

### Building a Multidisciplinary Team

Our project team consisted of EM faculty and residents, medical students, and undergraduates, with design assistance from members of a digital product studio at the UCSF School of Medicine, and topic expertise from physicians and social workers on our institution’s social medicine team. Hospital leadership, including the chief and vice chief of the Department of Emergency Medicine, were key stakeholders in the development and launch of the platform.

### Design Process

Our team used an iterative design-thinking approach to build, test, and deploy a homegrown digital decision tool called “Discharge Navigator” (edrive.ucsf.edu/dcnav). The design process occurred over a period of 18 months, beginning with interviews of key stakeholders (patients, clinicians, nurses, and social workers) and problem definition. Throughout this process, our team learned that existing platforms in the community resource arena did not meet our local needs; so we embarked on designing our own tool. In coordination with a digital product studio at the UCSF School of Medicine, we spent over 80 hours testing a series of concepts and prototypes with focus groups of EM residents and faculty. We learned that given the time constraints of medical practice, users preferred information to be displayed by relevance to their patient’s characteristics, rather than sorting through a long list of resources themselves. We also learned that users had particular difficulty recalling the eligibility requirements and pandemic restrictions for various resources, and designs in which these were prominently highlighted were more favorably received. To maximize ease of use, we ultimately decided to build a web-based tool housed within a larger digital hub designed for daily use by our staff and accessible via the electronic health record (EHR) interface.^8^

We asked our focus groups to brainstorm and rank social resource domains, determining that housing and substance use treatment resources would be the highest impact pilot modules. The resident physicians and medical students on our team conducted in-depth interviews with topic experts from our institution’s social medicine team^6^ to identify relevant resources and key branch points in the decision trees based on patient-related inputs. We filtered resource outputs based on acuity of care required, breadth of services required, and relevant patient demographic information (eg, primary language, gender, sexual orientation, pregnancy status, and age).

### Tool Development

**Figure 1:**
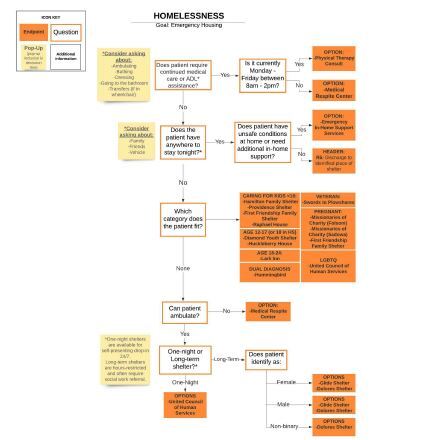
Decision tree for community resources to address homelessness, based on patient characteristics.

An example of the decision tree for resources for patients experiencing homelessness is included below and was developed using LucidChart^9^ (Lucid Software Inc, South Jordan, UT) (Figure 1). Once the decision trees and resource end points were finalized, a volunteer team of undergraduate and medical students developed a database of community resources under the guidance of resident physicians. This database includes standardized input fields for each resource’s hours and contact information, eligibility restrictions, insurance requirements, disability accessibility, interpreter services, duration of stay, current pandemic-related restrictions and protocols, and clinician actions necessary for referral. The team contacted each community partner by phone to verify information. Updates are conducted quarterly and tracked via a rigorous change-control document.

**Figure 2:**
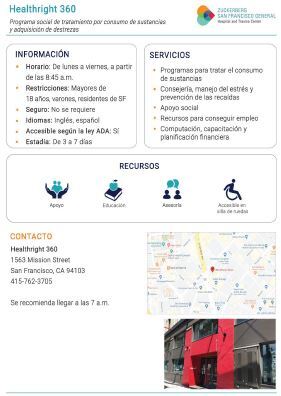
Example handout for a community substance use treatment center, in Spanish.

**Figure 3:**
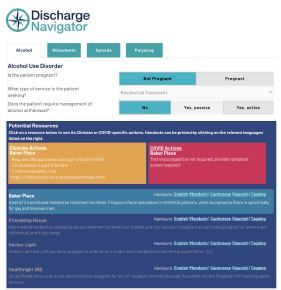
Sample of digital decision tool interface, with inputs and outputs.

Following the development of this database, our design team converted the standardized inputs for each resource into templated, single-page patient handouts (Figure 2). Handouts were translated from English into Spanish, Mandarin, Tagalog, and Cantonese by a private organization. We then converted the decision-tree algorithms and resource information into an intuitive and interactive digital decision tool called “Discharge Navigator,” using the web application development platform Bubble.io (New York, NY).^10^ Following the embedded decision-tree logic, the calculator-like interface translates patient-related inputs into a dynamic list of relevant resources, updating with each click (Figure 3). For each resource listed, the digital tool highlights any clinician action items needed to complete the referral, as well as any pandemic-related requirements such as necessary COVID-19 testing. Additionally, with each resource, the Discharge Navigator provides links to patient handouts in five different language options.

### Implementation and Evaluation

We built upon an institutional collaboration to create a digital tool for streamlining care in the COVID-19 pandemic^11^ and housed Discharge Navigator in our departmental digital resource hub, linked directly from our EHR system (Epic Systems Corporation, Madison, WI). We performed walk-throughs of the tool at departmental faculty and resident meetings, created a promotional video, and posted information flyers around the department.

In a 10-week period after platform launch, we conducted a single, anonymous, cross-sectional survey of emergency clinicians that asked them to recall their knowledge and confidence prior to deployment and compare that with the current state. We used Qualtrics (Provo, UT),^12^ with approval from our institutional review board. We considered previously validated survey measures whenever possible (eg, for perceived usefulness^13^ and usability^14^ of the digital tool) and adapted questions in the domains of tool understandability, navigability, ease of use, usefulness, and frequency of use to create a novel unvalidated survey (Supplement 1). We compared clinician knowledge and confidence pre- and post-implementation using chi-square statistical tests, ranked perceived barriers to referral, and measured tool usage and satisfaction metrics. Collecting clinician feedback enabled the project team to iteratively improve the usability of the tool and add an additional resource domain, mental health, upon completion of the pilot.

## IMPACT

**Figure 4:**
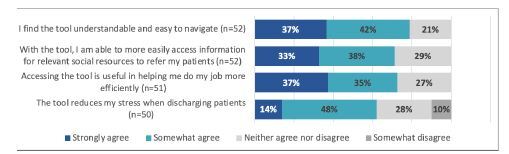
Impact of digital decision tool on clinician knowledge of and confidence in accessing homelessness and substance use resources.

**Figure 5:**
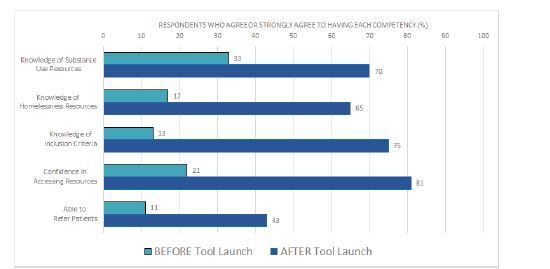
Clinician perceptions of the digital decision tool’s usability and usefulness.

During the study period, between 4–81 (average 23) individual IP addresses accessed the Discharge Navigator website per week. Fifty-five respondents completed the survey (response rate of 48%). Respondents were 58% residents and fellows, 34% attendings, and 8% nurse practitioners. Prior to the implementation of this tool, top cited barriers to referring patients to social resources were lack of knowledge of resources (44% ranked first), eligibility requirements (74% ranked first or second), and pandemic-related restrictions (20% ranked first). The launch of our tool yielded a statistically significant increase in awareness of homelessness and substance use resources, confidence in accessing resources, knowledge of eligibility criteria, and ability to refer patients always or most of the time (Figure 4). The majority of respondents found the tool useful and easy to navigate (Figure 5). We found that 53% of respondents used the tool one or more times per week, 89% used it at least once per month, 86% planned on using it more frequently, and 80% endorsed using the tool most often during nights and weekends. The average likelihood to recommend the tool to other clinicians was 7.8 of 10.

## DISCUSSION

We successfully designed, built, and implemented a custom digital decision tool for social discharge resources, which was regularly used by clinicians in a public tertiary ED. Importantly, our results suggest that Discharge Navigator is an effective educational tool for emergency clinicians at our institution. Our tool significantly increased self-reported clinician knowledge and confidence in referring patients to community resources for substance use treatment and housing insecurity. In effect, the tool may help directly address the most-cited clinician-specific barriers identified in our problem-definition interviews.

Our design process and implementation yielded several valuable insights that may assist in the development of similar tools at other institutions. We recommend first identifying current gaps and barriers to addressing patient social needs and identifying key stakeholders including supportive leadership. It is particularly effective to develop a multidisciplinary team that includes clinicians, social workers, designers, students, and patients. A design-thinking approach or gap analysis can help identify whether the appropriate intervention is a new vs existing tool.^15^ In busy practice settings in which changes to workflow can face resistance, designing with user input from the start can improve resultant adoption and satisfaction. Iterating our tool with the assistance of emergency clinician focus groups helped yield a product tailored for ease of use, with a high likelihood-to-recommend score and a large majority of users planning on increasing their use of the tool in their future workflows. Collecting clinician feedback also enabled our project team to iteratively improve the usability of the tool and add an additional resource domain, mental health, upon completion of the pilot.

It is important to consider project sustainability throughout the design process. Ensuring updated community resource information was our largest implementation hurdle, as it required regular, occasionally time-intensive interactions with community partners. We partnered with students from a volunteer organization with an aligned social mission (California Social Resource Database: caliresources.org), allowing for sustainability of future updates. A $5,000 portion of a local grant was also necessary to develop and implement this tool, including fees for our handout design and translation services. For practice settings in which additional funds are unavailable, it may be more difficult to offer patient resources in multiple languages. In addition, we encountered minor technical hurdles during the iterative tool buildout process (for example, while Bubble.io offers a user-friendly interface for updates, it is limited in its pre-set options for result filtration based on multiple patient inputs). This type of technical trade-off is important to consider when selecting a digital platform.

Our tool is a valuable addition to the existing literature of innovations to help better address social needs in the ED. Complementing prior work that describes dedicated care teams or clinics that bridge patients to resources,^6^,^15^–^16^ digital interventions require fewer resources and may be more feasible to implement in certain practice settings.^17^–^19^ There have been several published educational interventions to improve physician and nurse knowledge surrounding social medicine topics relevant to ED discharge, commonly in the form of modules, protocols, or EHR dot phrases.^18^,^20^ To our knowledge, Discharge Navigator is distinctive as an educational intervention for several reasons, including that it is freely accessible outside of the EHR (as well as easily linked within an EHR toolbar); spans multiple topic domains, and is designed for seamless addition of new modules; is interactive and customizable in real time to filter for specific patient characteristics (including vulnerable subgroups and treatment needs); highlights specific clinician actions for each resource; and offers simple, templated patient handouts in five languages (in contrast to discharge handouts with more complex content or heterogenous design^21^).

## LIMITATIONS

There are several limitations of this pilot study. Our cross-sectional analysis is based on self-reported metrics rather than objective measures, introducing the possibility of recall bias or inaccurate self-assessments.^22^–^23^ Using a retrospective pre/post assessment may have helped to limit response shift bias.^24^–^25^ The survey contained abbreviated or adapted questions rather than entire validated instruments. Given that our tool is custom-built for our practice setting, external validity is uncertain, although we believe that similar tools could easily be replicated and tested in other institutions based on our open-access model. Most importantly, while our pilot shows promising impact on emergency clinicians, the main limitation of our evaluation is the lack of direct patient outcomes. Survey respondents self-reported a significant increase in their ability to refer patients to resources, but there is not currently a process in which we can track the number of patients who follow through with referrals to third-party resources, as has been done in the evaluation of other types of interventions to increase social resource referrals from the ED.^17^–^19^ This is an important area of focus for future development, as our ultimate aim is for interventions such as this one to translate into tangible patient impact.

## CONCLUSION

We describe a replicable and innovative tool for improving the ability of clinicians to connect their patients with community resources, with demonstrable educational impact. By describing our design process, outcomes, and learnings, we hope that Discharge Navigator and similar tools may help build a community of emergency clinicians who regularly incorporate social determinants of health into their patient care.

## Supplementary Information
