# Supplementary material for: Discharge Navigator: Implementation and Cross-Sectional Evaluation of a Digital Decision Tool for Social Resources upon Emergency Department Discharge
Source: West J Emerg Med. 2022 Aug 11;23(5):637–43. doi: 10.5811/westjem.2022.5.55015 (PMC9541983; doi:10.5811/westjem.2022.5.55015)
Supplement: Supplementary file 1 [file wjem-23-637-s001.docx]

**Supplement.** The list of questions and available responses in the cross-sectional survey administered to respondents about their perceptions of the Discharge Navigator tool.

Q2.1 If you would like to take this survey, click the “Agree” button to start the survey.

- Agree

Q2.2 PRIOR TO the launch of the Discharge Navigator tool, I was aware of the breadth of services available in these areas for my patients:

|  | Strongly agree | Somewhat agree | Neither agree nor disagree | Somewhat disagree | Strongly disagree |
| --- | --- | --- | --- | --- | --- |
| Substance Use Resources | o | o | o | o | o |
| Housing Resources | o | o | o | o | o |

Q2.3 PRIOR TO the launch of the Discharge Navigator tool, I felt confident in accessing information for relevant social resources to refer my patients:

- Strongly agree
- Somewhat agree
- Neither agree nor disagree
- Somewhat disagree
- Strongly disagree

Q2.4 PRIOR TO the launch of the Discharge Navigator tool, I felt confident that I knew the inclusion & exclusion criteria that make patients good candidates for these social resources:

- Strongly agree
- Somewhat agree
- Neither agree nor disagree
- Somewhat disagree
- Strongly disagree

Q2.5 PRIOR TO the launch of the Discharge Navigator tool, I was able to refer my patients to the appropriate services based on their needs:

- Always
- Most of the time
- About half the time
- Sometimes
- Never

Q2.6 Please rank the main barrier(s) you faced in referring patients to social resources (substance use, mental health, housing etc) PRIOR TO the launch of the Discharge Navigator tool. (Rank from 1-8, with 1 being the highest priority):

 ______ Knowledge of COVID Restrictions

______ Knowledge of Eligibility Requirements for Resources

______ Knowledge of Resources

______ Lack of Relevant Informational Materials to Provide Patients

______ Language Barrier (of Provider or Resources)

______ Not in my job description as a clinician

______ Time

______ Other

Q2.7 SINCE THE LAUNCH of the Discharge Navigator tool, I am now familiar with the following types of resources available to patients in San Francisco:

|  | Strongly agree | Somewhat agree | Neither agree nor disagree | Somewhat disagree | Strongly disagree |
| --- | --- | --- | --- | --- | --- |
| Substance Use Resources | o | o | o | o | o |
| Housing Resources | o | o | o | o | o |

Q2.8 SINCE THE LAUNCH of the Discharge Navigator tool, I feel confident in accessing information for relevant social resources to refer my patients:

- Strongly agree
- Somewhat agree
- Neither agree nor disagree
- Somewhat disagree
- Strongly disagree

Q2.9 SINCE THE LAUNCH of the Discharge Navigator tool, I feel confident that I know the inclusion & exclusion criteria that make patients good candidates for these social resources:

- Strongly agree
- Somewhat agree
- Neither agree nor disagree
- Somewhat disagree
- Strongly disagree

Q2.10 SINCE THE LAUNCH of the Discharge Navigator tool, I am able to refer my patients to the appropriate services based on their needs:

- Always
- Most of the time
- About half the time
- Sometimes
- Never

Q2.11 For each of the following statements, please indicate how much you agree or disagree:

|  | Strongly agree | Somewhat agree | Neither agree nor disagree | Somewhat disagree | Strongly disagree |
| --- | --- | --- | --- | --- | --- |
| I find the tool understandable and easy to navigate | o | o | o | o | o |
| With the tool, I am able to more easily access information for relevant social resources to refer my patients | o | o | o | o | o |
| Accessing the tool is useful in helping me do my job more efficiently | o | o | o | o | o |
| The tool reduces my stress when discharging patients | o | o | o | o | o |

Q3.2 I use the Discharge Navigator tool approximately:

- Never
- Once per month
- Once per week
- Multiple times per week
- Daily

Q3.3 I use the tool most often during (select all):

- Weekdays (9am - 5pm)
- Weeknights (after 5pm)
- Weekends

Q3.4 How do you foresee your use of the Discharge Navigator tool changing over the next month?

- I plan on using the tool more frequently
- I plan on using the tool with the same frequency
- I plan on using the tool less frequently

Q3.5 How likely are you to recommend the Discharge Navigator tool to a colleague?


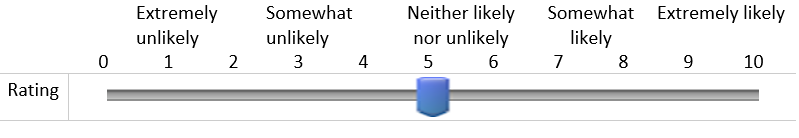


Q3.6 Which of the following domains would you like to see next on the Discharge Navigator tool? (Please rank from 1-12, with 1 being the highest priority)

______ Chronic Pain

______ Financial Security

______ Food Insecurity

______ Getting Insurance

______ Legal Aid Services

______ Home Care and Support

______ Interpersonal Violence

______ Primary Care

______ Sexual Health

______ Social Support

______ Transportation

______ Other

Q3.7 What would you like to see improved or added to the Discharge Navigator tool? (optional short answer)

________________________________________________________________

________________________________________________________________

________________________________________________________________
